# Core self-evaluation, mental health and mobile phone dependence in Chinese high school students: why should we care

**DOI:** 10.1186/s13052-022-01217-6

**Published:** 2022-02-14

**Authors:** Yun Li, Zhibin Wang, Weiquan You, Xiuqin Liu

**Affiliations:** Department of Psychological Evaluation and Intervention, Xiamen Xianyue Hospital, No. 399, Siming District, Xiamen, 361012 Fujian Province China

**Keywords:** Core self-evaluation, Mental health, Mobile phone dependence, High school, Student, Care

## Abstract

**Background:**

Mobile phone dependence is a common problem in the population of high school students. We aimed to evaluate the core self-evaluation, mental health and mobile phone dependence in Chinese high school students, to provide reliable evidence to the support of high school students.

**Methods:**

We conducted a survey of high school students in Xiamen, China. The self-assessment questionnaire on mobile phone use dependence among teenagers (SQAPMPU), Mental Health Scale for Middle School Students (MSSMHS) and Core self-evaluation scale were used to evaluate the mobile phone dependence, mental health and core self-evaluation. t-tests, Pearson correlation and multiple linear stepwise regression analyses were conducted to analyze the potential relationships.

**Results:**

A total of 1692 students were enrolled. The total score of mobile phone dependence of students in grades 10–12 was higher than that of students in grades 7–9. A total of 329 students in grades 7–9 and 371 students in grades 10–12 had abnormal mental status. The detection rate of psychological abnormalities among students in grades 10–12 was higher than that of students in grades 10–12. Core self-evaluation had significantly negative correlation with various factors of mental health (*P* < 0.01). The worse the mental health, the higher the degree of mobile phone dependence, core self-evaluation played a mediating role between mental health and mobile phone dependence (8.03%).

**Conclusions:**

Core self-evaluation is an important factor affecting the mental health of high school students and mobile phone dependence. Educators should strengthen core self-evaluation of high school students to improve the mental health and reduce the mobile phone dependence.

## Background

Mobile phone dependence refers to a state of uncontrollable fascination when an individual negatively uses a mobile phone for non-study or work purposes, accompanied by physical or psychological discomfort, which affects social functions [1]. According to reports [2, 3], the number of mobile phone netizens in China has reached 825 million, of which 29.24 to 66.14% of high school students have mobile phone dependence. Mobile phone dependence causes damage to the physical and mental problems and social functions of high school students, such as depression, sleep problems, and poor cognitive and reduced learning abilities [4, 5]. Therefore, researches on mobile phone dependence are of great significance to the physical and mental health of high school students.

There are many factors that affect high school students’ mobile phone dependence, and core self-evaluation may be one of the important factors [6]. Core self-evaluation is a potential and broad personality structure [7]. It is the most basic evaluation of an individual’s self-ability and value, which associated with different kind of dependence or addiction. However, as an important influencing factor of mobile phone dependence, low core self-evaluation does not necessarily lead to mobile phone dependence of high school students. Previous study has found that core self-evaluation can negatively predict anxiety [8]. Individuals with high core self-evaluation are full of confidence in life, believe that they have a good sense of control over most things in life, and less anxiety due to lack of control [9]. Anxiety refers to an unpleasant inner conflict that an individual has in the face of an upcoming event that may pose a threat, accompanied by certain emotional and physical symptoms, such as anger, tension, insomnia, and stomach pain [10]. Previous studies [11, 12] have shown that anxiety can positively predict mobile phone dependence. Anxious individuals believe that they can relax themselves when using mobile phones, so that they can temporarily stay away from anxious events and environments, and gain a sense of control over themselves [13]. However, those are often the first sign and feelings of mobile phone dependence. We hypothesize that individuals with mobile phone dependence may have poorer core self-evaluation that gradually development to the mobile phone dependence. Therefore, we aimed to evaluate the role of core self-evaluation in the mobile phone dependence and mental health of high school students, to provide insights to the management and care of physical and mental health of high school students.

## Methods

In this study, all methods were performed in accordance with the relevant guidelines and regulations. This survey protocol had been verified and approved by the ethical committee of our hospital (approval number: 2019-KY-016). And written informed consent was obtained from all the included participants.

### Study populations

A stratified random sampling method was adopted in this present study. In June 2020, three classes were randomly selected from each grade of the 4 general high schools in Xiamen City, a total of 36 classes were included finally.

### Survey tools

#### The self-assessment questionnaire on mobile phone use dependence among teenagers (SQAPMPU)

The SQAPMPU [14] was developed by Tao Fangbiao, Department of Child and Adolescent Hygiene and Maternal and Child Health Sciences, School of Public Health, Anhui Medical University, in 2012, The questionnaire has a total of 13 items, and it is divided into three dimensions: withdrawal symptoms, cravings, and physical and mental effects. The higher the total score, the higher the degree of dependence on mobile phones. The Cronbach’s a coefficient of the total questionnaire is 0.87, and the Cronbach’s a coefficient of each dimension is above 0.5, and the interval is 0.58–0.83.

#### Mental health scale for middle school students (MSSMHS)

The scale was developed by Professor Wu [15] and can be used to assess the mental health of high school students. The scale has 60 items and 10 subscales, including obsessive-compulsive symptoms, paranoia, hostility, interpersonal sensitivity, depression, anxiety, learning pressure, maladjustment, mood swings, and psychological imbalance. The test-retest reliability of the subscale is 0.716–0.905, the homogeneity reliability is 0.6010–0.8577, and the split-half reliability is 0.6341–0.8726. The correlation coefficient between the subscale and the total scale is 0.7652–0.8726, which has good construct validity. The subjects would conduct self-evaluation on the true situation of their recent psychological state. The scale is five point likert scale, and each item is a declarative sentence. The total score ≥ 2 points is regarded as abnormal mental health detection. The total score and subscale scores of 2–2.99 points are taken as mild abnormalities, 3–3.99 points are taken as moderate abnormalities, 4–4.99 are taken as severe abnormalities, and 5 points are taken as severe abnormalities.

#### Core self-evaluation scale

This scale is a tool to directly measure core self-evaluation compiled by Zenger et al. [16]. Based on relevant theories and research, Du et al. [17] translated and revised the core self-evaluation scale, and formed the core self-evaluation scale under the Chinese cultural background. The core self-evaluation is a one-dimensional self-evaluation scale consisting of 10 items, using a five-level score, ranging from 1 to 5 to indicate complete disagreement to complete agreement. The total score ranges from 10 to 50 points. The higher the score, the higher the core self-evaluation level. The Cronbach’s alpha coefficient of the core self-evaluation scale is 0.83, the split-half reliability is 0.84, and the test-retest reliability of the 3-week interval is 0.82.

### Data collection

A total of four nurses from our hospital formed a research team. After standardized training, they were randomly distributed to school to conduct relevant research. During the survey, every two researchers formed a group to complement and check each other. Each item of the questionnaire in the survey was queried and filled out by the respondents. It might need 15 ~ 25 min to finish every survey.

### Statistical methods

SPSS 23.00 was used for data analysis. Post hoc analysis for sample size was performed to analyze the power of the study. Independent sample t-tests were performed on the differences on the students’ mobile phone dependence status, mental health status, and core self-evaluation of high school students. A Bonferroni correction for multiple comparisons was applied. Pearson correlation analysis and multiple linear stepwise regression analysis were carried out on the mobile phone dependence status, mental health status and core self-worth of high school students. The structural equation model was established with STATA to test the mediation effect. In this study, the inspection level was set as α = 0.05 with two-sided inspection.

## Results

### The characteristics of included students

A total of 1692 students were enrolled in this survey, 1692 questionnaires were distributed, and 1637 valid questionnaires were returned. The effective rate of the questionnaire distribution was 96.75%. There are 832 students in grades 7–9 with an average age of 14.63 ± 1.22 years old, and 805 students in grades 10–12 with an average age of 17.45 ± 1.53 years old.

### The mobile phone dependence status

As shown in Table 1, students in grades 10–12 had significantly higher physical and mental scores than that of students in grades 7–9 (*P* < 0.01). There was no significant difference in withdrawal symptoms and craving behavior (*P* > 0.05). The total score of mobile phone dependence of students in grades 10–12 was higher than that of students in grades 7–9, and there was a statistical difference (*P* < 0.05).

**Table 1:** The mobile phone dependence status of high school students (±s)

	Students in grades 7–9 (*n* = 832)	Students in grades 10–12(*n* = 805)	*t*	*P*
Withdrawal symptoms	10.28 ± 4.971	10.31 ± 4.092	−0.109	0.913
Craving behavior	5.02 ± 2.177	5.03 ± 1.958	−0.123	0.902
Physical and mental influence	7.38 ± 3.533	8.41 ± 3.379	−6.02	0.000**
Total score	22.68 ± 9.574	23.75 ± 8.203	−2.415	0.016*

*P < 0.05, **P < 0.01

### The mental health status

When the total score of MSSMHS ≥2 points, it was regarded as abnormal mental health. As shown in Table 2, a total of 503 (60.5%) of students in grades 7–9 with normal mental health, a total of 329 students in grades 7–9 and 371 students in grades 10–12 had abnormal mental status. The detection rate of psychological abnormalities among students in grades 10–12 was higher than that of students in grades 7–9(*P* = 0.000).

**Table 2:** The mental health status of high school students

	Normal	Mild abnormal	Moderate abnormal	Severe abnormal	Total abnormal
Students in grades 7–9 (*n* = 832)	503 (60.5)	245 (29.4)	71 (8.5)	13 (1.6)	329 (39.5)
Students in grades 10–12(*n* = 805)	434 (53.9)	307 (38.1)	62 (7.7)	2 (0.2)	371 (46.1)
χ^2^	20.281				
P	0.000				

As shown in Table 3, the scores of students in grades 10–12 in learning stress, maladaptaion, and mood swings were significantly higher than those of students in grades 10–12 (all *P* < 0.01). There were no significant differences in the scores of obsessive-compulsive symptoms, paranoid, hostility, interpersonal sensitivity, depression and anxiety between students in grades 7–9 and students in grades 10–12 (*P* > 0.05).

**Table 3:** Analysis of differences in mental health status of high school students

	Students in grades 7–9 (*n* = 832)	Students in grades 10–12(*n* = 805)	*t*	*P*
Obsessive-compulsive symptoms	2.12 ± 0.744	2.12 ± 0.657	−0.097	0.923
Paranoid	1.87 ± 0.828	1.89 ± 0.75	−0.354	0.723
Hostility	1.8 ± 0.872	1.76 ± 0.742	1.187	0.235
Interpersonal sensitivity	1.98 ± 0.839	2.04 ± 0.754	−1.495	0.135
Depression	2.00 ± 0.948	2.02 ± 0.833	−0.664	0.507
Anxiety	2.08 ± 1.025	2.13 ± 0.902	−1.077	0.282
Feeling of learning pressure	2.08 ± 0.925	2.22 ± 0.842	−3.304	0.001**
Maladaptation	1.76 ± 0.696	1.98 ± 0.697	−6.319	0.000**
Mood swings	2.09 ± 0.830	2.22 ± 0.739	−3.361	0.001**
Psychological imbalance	1.62 ± 0.648	1.69 ± 0.622	−2.340	0.019*

*P < 0.05, **P < 0.01

### Core self-evaluation score

As indicated in Table 4, there was no significant difference in core self-evaluation score between students in grades 7–9 and students in grades 10–12(*P* = 0.298).

**Table 4:** Analysis of differences in core self-evaluation of junior and senior high school students

	Students in grades 7–9 (*n* = 832)	Students in grades 10–12(*n* = 805)	*t*	*P*
Core self-evaluation score	34.52 ± 7.437	34.15 ± 7.053	1.042	0.298

### Correlation analysis of mobile phone dependence, mental health and core self-evaluation

As shown in Table 5, Pearson’s correlation analysis indicated that core self-evaluation had a significant negative correlation with mobile phone dependence and various factors (*P* < 0.01); mental health status and various factors have a positive correlation with mobile phone dependence and various factors (*P* < 0.01). Among them, the correlation coefficients between learning pressure and mobile phone dependence and various factors are the most significant, respectively 0.555, 0.509, 0.471, 0.477. Core self-evaluation had a significant negative correlation with various factors of mental health (*P* < 0.01).

**Table 5:** Pearson correlation analysis of mobile phone dependence, mental health and core values of high school students

	Core self-evaluation	Withdrawal symptoms	Craving behavior	Physical and mental influence	Total score of mobile phone dependence
Core self-evaluation	1	−0.357^**^	−0.287^**^	− 0.371^**^	−0.394^**^
Obsessive-compulsive symptoms	−0.430^**^	0.368^**^	0.301^**^	0.343^**^	0.391^**^
Paranoid	−0.478^**^	0.463^**^	0.406^**^	0.411^**^	0.491^**^
Hostility	−0.482^**^	0.475^**^	0.414^**^	0.408^**^	0.497^**^
Interpersonal sensitivity	−0.554^**^	0.468^**^	0.392^**^	0.461^**^	0.509^**^
Depression	−0.660^**^	0.463^**^	0.380^**^	0.429^**^	0.492^**^
Anxiety	−0.619^**^	0.463^**^	0.373^**^	0.421^**^	0.487^**^
Feeling of learning pressure	−0.487^**^	0.509^**^	0.471^**^	0.477^**^	0.555^**^
Maladaptation	−0.417^**^	0.442^**^	0.412^**^	0.444^**^	0.495^**^
Mood swings	−0.536^**^	0.460^**^	0.396^**^	0.473^**^	0.511^**^
Psychological imbalance	−0.336^**^	0.445^**^	0.421^**^	0.402^**^	0.481^**^

*P < 0.05, **P < 0.01

### Regression analysis

Taking various factors of mental health status and core self-evaluation as independent variables, and taking the total of mobile phone dependence as dependent variables, a multiple linear stepwise regression analysis was performed. As indicated in Table 6, the results showed that feeling of learning pressure, interpersonal sensitivity, psychological imbalance, core self-evaluation, hostility, obsessive-compulsive symptom and maladaptation had a significant influence on the mobile phone dependence (all *P* < 0.05).

**Table 6:** Regression analysis on the influencing factors of mobile phone dependence in high school students

	β	Standard error	t	*P*
Constant	14.285	1.481	9.647	0.000
Feeling of learning pressure	2.904	0.294	9.863	0.000
Interpersonal sensitivity	0.942	0.415	2.269	0.023
Psychological imbalance	1.824	0.425	4.295	0.000
Core self-evaluation	−0.128	0.030	−4.282	0.000
Hostility	1.331	0.345	3.857	0.000
Obsessive-compulsive symptoms	−1.163	0.371	−3.137	0.002
Maladaptation	1.212	0.393	3.084	0.002

### Analysis of the mediating role of core self-evaluation between mental health status and mobile phone dependence

**Fig. 1:**
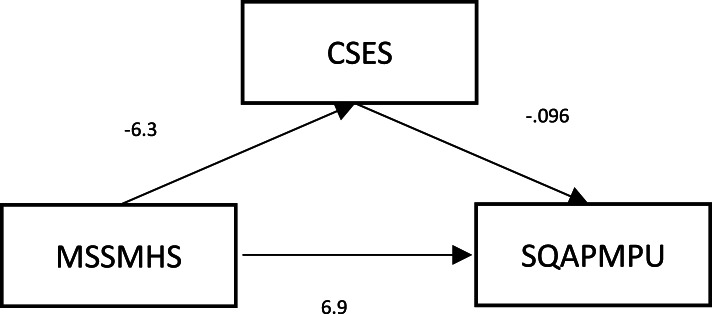
The chart of the mediating role of core self-evaluation between mental health status and mobile phone dependence. Notes: mssmhs, mental health status; sqapmpu, mobile phone dependence; cses, core self-evaluation secore

We used mental health status as the independent variable, mobile phone dependence as the dependent variable, and core self-evaluation as the intermediary variable for structural equation modeling. As shown in Fig. 1 and Table 7, the direct effect of mental health status on mobile phone dependence was 6.949 (*P* = 0.000), the indirect effect was 0.607, and the total mediating effect was 8.03%. The results showed that mental health had a significant negative direct predictive effect on mobile phone dependence. The worse the mental health, the higher the degree of mobile phone dependence, core self-evaluation played a mediating role between mental health and mobile phone dependence.

**Table 7:** Analysis of the mediating role of core self-evaluation between mental health status and mobile phone dependence

	Direct effect		Indirect effect		Mediation effect
	Coefficient(95%CI)	*P*	Coefficient(95%CI)	*P*	
Core self-evaluation	6.949 (6.305, 7.593)	0.000	0.607 (0.221, 0.992)	0.000	8.03%

## Discussions

High school students are in a conflicting period of self-identity and role confusion. When their ideal self is inconsistent with their real self, they feel pain and anxiety, looking for ways to alleviate their inner anxiety [18]. Mobile phones have the characteristics of versatility, easy availability, and convenience, and it has become their first choice tool for dispelling depression and anxiety [19]. There are significant differences in anxiety and core self-evaluation among high school students in the mobile phone dependent group and the non-mobile phone dependent group. On the one hand, the anxiety score of the mobile phone dependent group is higher than that of the non-mobile phone dependent group. Because the mobile phone dependent students invest in mobile phone dating, entertainment, online shopping and other activities, they lack face-to-face communication [20, 21]. However, human beings are a kind of social animals. If they stay in the virtual world for a long time, they will have a sense of discomfort in real life, and anxiety will follow [22]. On the other hand, the core self-evaluation of mobile phone dependents is lower than those of non-mobile phone dependents, because students’ activities are mainly interpersonal communication and learning, while mobile phone dependents often have high levels of negative emotions, low feelings of social support, high learning burnout [23, 24]. For problems such as low learning efficiency, they will have a lower self-evaluation of their abilities and value.

Individuals with high core self-evaluation have less stress and tension in life and study, have stronger control, and are more efficient in study [25]. They often use emotion regulation to balance the body, and use less avoidance strategies such as playing mobile phones to cope [26]. Highly anxious individuals have a large gap between themselves and reality. They will find some way to reduce this gap or escape from reality [27]. Since the characteristics of mobile phones are just in line with their needs, they will spend a lot of time on mobile phones [28]. It is difficult to control oneself, and the withdrawal reaction may occur after being isolated from the mobile phone for a period of time, so the dependence on the mobile phone is high in high school students [29]. The core self-evaluation of students has a negative predictive effect on mobile phone dependence behavior, which is consistent with the hypothesis of this research [30]. Core self-evaluation is a positive personality structure that can effectively reduce students’ mobile phone dependence behavior, make mobile phones an aid in their lives and studies, and make scientific and reasonable arrangements for mobile phone use [31]. However, the prediction of anxiety on mobile phone dependence is positive. Worry and anxiety will not reduce the individual’s use of mobile phones [7]. On the contrary, the more anxiety, the more mobile phone dependence will be generated [32].

Core self-evaluation plays a mediating role between mental health and mobile phone dependence. Motivations for mobile phone use include interpersonal communication, information acquisition, self-expression, and leisure and entertainment [33]. When high school students’ mobile phone motivation is high, it means that real life can no longer meet their needs in these four areas [33]. In this case, mobile media provides a relatively safe environment, which enables high school students to have maximum control over the use of mobile phones. The social functions of mobile phones can compensate for the desire for interpersonal relationships with high neuroticism, low rigor, and high openness [34, 35]. For example, high school students with neurotic personality may have obstacles to interpersonal communication due to emotional instability. Mobile phones can make them avoid contact with others to the greatest extent, and in this way can compensate for the lack of information obtained due to the narrow social network [36, 37]. The anonymity of using social software on mobile phones can enable them to escape the negative emotions in real life, and moderate use of mobile phones can regulate negative emotions and obtain a sense of happiness [38, 39]. Besides, using the extensive functions of mobile phones for entertainment can make individuals temporarily divert their attention from stress and competition and avoid problems [40, 41]. However, when the motivation to use such evasive mobile phones is too strong, the frequency and time of mobile phone use will increase, and ultimately causes mobile phone dependence, resulting in mental health problems [42]. Because of the high motivation for mobile phone use, the level of pleasure and seriousness of users is higher than those with low motivation for mobile phone use [43, 44]. For students with moody, highly neurotic personality, the unorganized low-rigorous personality, and the highly open personality who likes new things, the degree of their addiction to mobile phones is more important, and the emergence of mental health problems becomes inevitable [45, 46]. Additionally, students who frequently use mobile phones and are highly motivated to use mobile phones can also use mobile phones to understand the privacy of their close peers and monitor their peers’ behaviors, so as to meet their peer needs and sense of belonging [47]. This can also affect the formation of the intermediary variable mobile phone addiction tendency.

Several limitations in this present study must be considered. Firstly, no physician or psychologist has assessed the included subjects in this study, there can be biased during the survey process of different health care providers. Secondly, although the results of post hoc analysis for sample size have showed enough power of the study, limited by resource, the sample size of this study is not big enough, which may underpower to detect the potentially influencing factors. Thirdly, there are many differences in the education systems amongst different countries. Chinese educative system tends to be more stricter, some high school in China may have the rule of not allowing telephone use during school stay. Therefore, some findings in this present study may not be generalized to different systems in other countries. Thirdly, this study is a cross-sectional survey, and we have not conducted a long-term follow-up survey of students’ dependence behavior. Future studies with larger sample size should be conducted longitudinally to identify the long-term influences of core self-evaluation in mediating mental health and mobile phone dependence.

## Conclusions

In conclusion, our survey provides certain theoretical guidance for high school students’ mobile phone dependence behavior, we have found that core self-evaluation has a certain influence on college students’ mobile phone dependence and health status. Improving high students’ core self-evaluation may be beneficial to improve the health status and reduce mobile phones dependence.

## Data Availability

All data generated or analyzed during this study are included in this published article.

## Abbreviations

SQAPMPU: The self-assessment questionnaire on mobile phone use dependence among teenagers
MSSMHS: Mental Health Scale for Middle School Students
